# Arsenic Transformation in Soil-Rice System Affected by Iron-Oxidizing Strain (*Ochrobactrum* sp.) and Related Soil Metabolomics Analysis

**DOI:** 10.3389/fmicb.2022.794950

**Published:** 2022-02-21

**Authors:** Ziyan Qian, Chuan Wu, Weisong Pan, Xiaoran Xiong, Libing Xia, Waichin Li

**Affiliations:** ^1^College of Bioscience and Biotechnology, Hunan Agricultural University, Changsha, China; ^2^School of Metallurgy and Environment, Central South University, Changsha, China; ^3^Department of Science and Environmental Studies, The Education University of Hong Kong, Tai Po, Hong Kong SAR, China

**Keywords:** arsenic, iron-oxidizing bacteria, paddy soil, metabolomics, microbial community

## Abstract

Iron-oxidizing bacteria (FeOB) could oxidize Fe(II) and mediate biomineralization, which provides the possibility for its potential application in arsenic (As) remediation. In the present study, a strain named *Ochrobactrum EEELCW01* isolated previously, was inoculated into paddy soils to investigate the effect of FeOB inoculation on the As migration and transformation in paddy soils. The results showed that inoculation of *Ochrobactrum* sp. increased the proportion of As in iron-aluminum oxide binding fraction, which reduced the As bioavailability in paddy soils and effectively reduced the As accumulation in rice tissues. Moreover, the inoculation of iron oxidizing bacteria increased the abundance of KD4-96, *Pedosphaeraceae* and other bacteria in the soils, which could reduce the As toxicity in the soil through biotransformation. The abundance of metabolites such as carnosine, MG (0:0/14:0/0:0) and pantetheine 4’-phosphate increased in rhizosphere soils inoculated with FeOB, which indicated that the defense ability of soil-microorganism-plant system against peroxidation caused by As was enhanced. This study proved that FeOB have the potential application in remediation of As pollution in paddy soil, FeOB promotes the formation of iron oxide in paddy soil, and then adsorbed and coprecipitated with arsenic. On the other hand, the inoculation of *Ochrobactrum* sp. change soil microbial community structure and soil metabolism, increase the abundance of FeOB in soil, promote the biotransformation process of As in soil, and enhance the resistance of soil to peroxide pollution (As pollution).

## Introduction

Arsenic (As) is the most widespread toxic non-metallic element in nature, among which inorganic As (iAs) was identified as a class I carcinogen by the International Agency for Research on Cancer (IARC). As mainly exists as a sulfide (FeAsS, As_4_S_4_, and AsS) or is associated with other metal minerals in the natural environment. As mining and smelting, pesticide production, coal combustion and other human activities are important causes of environmental As pollution, and according to statistics, the global anthropogenic emissions of As in soil are 2.84 × 10^5^–9.4 × 10^5^ t (Clemens and Ma, 2016). According to a survey conducted by the Ministry of Environmental Protection and the former Ministry of Land and Resources, by 2014, the total exceeding rate of soil pollution in China had reached 16.1%, while the exceeding rate of soil sampling sites on cultivated land was 19.4%, and the exceeding rate of As samples was 2.7%, ranking third among the eight inorganic pollutants after cadmium and nickel. Rice is the staple food for approximately 3 billion people worldwide (Zhao et al., 2015). Pollution in paddy soil not only causes plant poisoning and crop yield loss but also enters the human body through the food chain, posing a serious threat to human health (Saifullah, et al., 2018). Therefore, the remediation of As-contaminated paddy soil is a major ecological and environmental problem, and it is urgent to find a cost-effective and environmentally friendly solution.

Soil microorganisms play an important role in affecting the geochemical cycling of arsenic, regulating its morphological transformation, environmental fate and bioavailability. Sforna et al. (2014) showed that microbial metabolism of As began 2.7 billion years ago, and some specific microorganisms contain active enzymes related to arsenic metabolism, which are able to regulate the redox and methylation processes of As in microbial cells (Sforna et al., 2014). Arsenic-oxidizing bacteria contain the arsenite oxidase gene (aioA), which can mediate arsenic oxidation, and their activities, such as in *Ensifer* sp. and *Acinetobacter* sp., lead to reduced toxicity of arsenic in soil and may help to stimulate the growth/activity of indigenous microorganisms in soil and promote the remediation of arsenic-contaminated soil by hyperaccumulator plants (Wang et al., 2012; Debiec-Andrzejewska et al., 2020). The genetically engineered bacteria *Pseudomonas putida* KT2440, *Bacillus subtilis*, and *Westerdykella aurantiaca* contain the As(III) *S*-adenosylmethionine methyltransferase gene (arsM) in their cells, which can methylate arsenic to methyl arsenate [MAs(V)], dimethyl arsenate [DMAs(V)] and trimethyl arsenic oxide [TMAs(V)O] to promote the production of volatile arsenic compounds, thereby reducing the inorganic arsenic content in soil (Chen et al., 2014; Verma et al., 2016). In addition, some other species of microorganisms have been reported to be involved in the remediation of As contamination. For example, the activity of sulfate-reducing bacteria and their subsequent production of sulfide precipitate As(III) or coprecipitate with iron can sequester As(III) in the mineral phase (Kirk et al., 2004; Alam and McPhedran, 2019). Achal et al. (2012) successfully reduced exchangeable As in soil and sequestered arsenic in carbonate precipitation through a calcite precipitation process induced by *Sporosarcina ginsengisoli*. *In situ* remediation of arsenic in soil by microorganisms has gradually become a popular research topic due to its advantages in regard to environmental disturbance and cost effectiveness (Achal et al., 2012).

The anaerobic oxidation capacity of FeOB offers the possibility for its potential application for effectively removing arsenic in soil and groundwater treatment. It was found that FeOB, including *Acidovorax* sp. strain BoFeN1 and *Rhodobaster ferrooxidans* strain SW2, strain KS has the ability to resist arsenic poisoning, and the oxidation of Fe(II) produces mineral phases such as goethite and nano goethite, which can effectively remove more than 96% of arsenic in solution (Hohmann et al., 2010). The rhizosphere soil environment is influenced by plant root exudates and rhizosphere microorganisms. Physicochemical properties of the soil rhizosphere (pH, Eh, EC, element concentration, etc.) and environmental factors (radial oxidation loss, metabolites secreted by plants, microbial activity, etc.) may affect the bioavailability of arsenic in paddy soil (Bais et al., 2006). Under flooded paddy soil, radial oxidation loss of rice roots induced the formation of iron plaque on the root surface (Wu et al., 2015), which sequestered As by adsorption or coprecipitation with iron oxides, thus affecting the bioavailability of As in the rhizosphere, serving as an important barrier against the uptake of As in rice (Tripathi et al., 2014). The flooding and Fe- and N-rich conditions in the paddy soil provided a suitable living environment for microaerobic nitrate-dependent FeOB (Ratering and Schnell, 2001). The microbial iron oxidation process successfully competes with chemical iron oxidation and occupies a dominant position. Nitrate-dependent FeOB utilizes nitrate as an electron acceptor to oxidize Fe(II) to Fe(III) (Klueglein et al., 2014; Jamieson et al., 2018) and mediates the metabolic pathway of denitrification and the reduction of alien nitrate to ammonia. It is the coupling link of the iron cycle and nitrogen cycle (Li et al., 2016) and plays a role in the biogeochemical cycle of As.

Several studies have proven that artificial inoculation of FeOB could stimulate the formation of iron plaque in rice roots and significantly reduce the concentration of As in rice roots, leaves, glumes and grains (Dong et al., 2016; Xiao et al., 2020). However, most studies focused on the effect of iron-oxidizing bacteria inoculation on arsenic fixation in soil and rice plants but paid little attention to the potential changes of microbial community structure and soil metabolism after iron-oxidizing bacteria inoculation. In this study, a new FeOB isolated in our previously study, *Ochrobactrum* sp. EEELCW01, was inoculated into paddy soil. The main objectives of this study were: (a) to explore the redistribution of arsenic in paddy soil and rice plants by inoculation with FeOB; (b) to investigate the changes of soil microbial community structure after inoculation with FeOB; (c) to understanding the regulatory mechanism of FeOB in soil from the perspective of metabonomics.

## Materials and Methods

### Tested Strain and Growth Conditions

*Ochrobactrum* EEELCW01, a bacterium that can oxidize ferrous iron under microaerobic conditions, was isolated from a typical arsenic-contaminated paddy soil in Furong District, Changsha city, Hunan Province (Luo et al., 2021). The accession numbers of this bacterium in the NCBI GenBank are CP047598 and CP047599. After activation, *Ochrobactrum* EEELCW01 was cultured in liquid LB medium in a rotary shaker at 28°C and 200 rpm. After 48 h of culture after inoculation, the bacteria grew to the logarithmic phase, and the medium was centrifuged at 8000 rpm to collect the precipitated cells, which were washed with neutral sterile water three times to prepare a resting cell suspension.

### Soil Source and Preparation of Pot Experiments

Pot experiment soils were collected from the surface (0–20 cm) of a paddy field near mining areas in Chenzhou, Hunan Province, China. Basic soil physical and chemical properties, including pH, EC, organic matter, available potassium, available nitrogen, total iron, aluminum, manganese and arsenic content, were determined, as shown in Table 1. To avoid affecting the composition of the soil microbial community and the abundance of arsenic metabolic genes, soil was immediately collected and used in pot experiments. The soil was packed in PVC pots (height 30 cm, bottom diameter 24 cm, and diameter 28 cm), and each pot contained 10 kg of soil. Fertilizer was applied according to the conventional fertilization ratio of N: P_2_O_5_:K_2_O = 1.5:1:2. In addition, 10 mmol/L sodium nitrate was added to supplement the additional nitrogen source for FeOB growth. The pot soil was irrigated with sterile water to reach field moisture capacity and equilibrated for approximately 1 week.

**TABLE 1:** Basic properties of the soil for pot experiments.

	pH	As (mg/kg)	Al (mg/kg)	Cd (mg/kg)	Cr (mg/kg)	Cu (mg/kg)	Fe (g/kg)	Mn (g/kg)	Pb (g/kg)	Zn (g/kg)
Soil	7.25	142.5	250.4	22.05	168.1	155.1	16.43	9.330	1.460	89.89

The rice seeds were soaked in 30% (H_2_O_2_) hydrogen peroxide solution for 15 min to sterilize the surface and then transferred into petri dishes covered with moist filter paper for germination. When the seedlings (2–3 cm) germinated, they were transferred into seedling raising trays paved with paddy soil for seedling raising. Three uniform 2-week-old seedlings were selected and transplanted into each soil-filled PVC pot (Wu et al., 2014).

### Pot Experimental Design

Four treatments were prepared in this study: (1) CK (controls without inoculation of bacteria and cultivation of rice plants), (2) FB (soil with inoculation of FeOB), (3) RP (soil with cultivation of rice plants) and (4) RF (soil with cultivation of rice plants and inoculation of FeOB on rice roots). Each treatment was replicated three times. To simulate actual field operations, rice seedlings were grown under flooded conditions (water surface 2 cm above the soil surface). The prepared bacterial suspension (100 mL/pot) was uniformly injected into rhizosphere soil by syringe.

The rhizosphere and non-rhizosphere soil solutions of rice plants were sampled with a Rhizon soil solution sampling tube (Rhizosphere, Netherlands) on Day 0, Day 15 (rice-tillering stage), Day 30 (rice-jointing stage), Day 45 (rice-heading stage), Day 75 (rice-filling stage) and Day 105 (rice-maturing stage) after seedling growth to analyze their pH, conductivity (EC), total As, available As content, As speciation [As(III) and As(V)] and iron (Fe) content (Wu et al., 2011; Yu et al., 2017). Soil samples were collected to determine the fractionation of As in the soil by sequential extraction procedures (Wenzel et al., 2001), and the available As was extracted by 0.5 mol⋅L^–1^ NaHCO_3_ (Woolson et al., 1971). Rice plants were harvested at the mature stage, washed with deionized water and then thoroughly rinsed with deionized water. After the surface moisture was air-dried, the arsenic content and arsenic species in the roots and straw were determined. In addition, at the maturity stage of rice, 50 mg solid soil samples were placed in a 1.5 mL centrifuge tube, quickly frozen in liquid nitrogen and immediately placed in a –80°C refrigerator for subsequent microbial diversity and metabolomics measurements.

### Determination of Arsenic Speciation in Rice Plant Parts

The washed rice plants were divided into root and straw parts, dried in a freeze-drying oven, and ground and crushed under liquid nitrogen for later use. A 0.2–0.5 g sample was weighed in a 50 mL centrifuge tube and 20 ml of 0.28 mol/L HNO_3_ solution was added and extracted in a 95°C water bath for 1.5 h. The extract was cooled to approximately 25°C and centrifuged at 5000 r/min for 10 min, and the supernatant was filtered through a 0.22 μm membrane for further analysis. The speciation content of arsenic in the samples was determined with hydride generation atomic fluorescence spectrometry (HG-AFS, AFS-8230, Beijing Jitian Instruments Co., Beijing, China).

### Microbial DNA Extraction and Illumina Sequencing

Microbial community genomic DNA was extracted from soil samples using the E.Z.N.A.^®^ soil DNA Kit (Omega Bio-tek, Norcross, GA, United States) according to the manufacturer’s instructions. The DNA extract was checked on a 1% agarose gel, and DNA concentration and purity were determined with a NanoDrop 2000 UV-vis spectrophotometer (Thermo Scientific, Wilmington, DE, United States). The hypervariable region V3-V4 of the bacterial 16S rRNA gene was amplified with primer pairs 338F (5′-ACTCCTACGGGAGGCAGCAG-3′) and 806R (5′-GGACTACHVGGGTWTCTAAT-3′) by an ABI GeneAmp^®^ 9700 PCR thermocycler (ABI, Los Angeles, CA, United States). PCR amplification of the 16S rRNA gene was performed as follows: initial denaturation at 95°C for 3 min, followed by 27 cycles of denaturing at 95°C for 30 s, annealing at 55°C for 30 s, extension at 72°C for 45 s, a single extension at 72°C for 10 min, and a final extension at 4°C. The PCR mixtures contained 5 × *TransStart* FastPfu buffer 4 μL, 2.5 mM dNTPs 2 μL, forward primer (5 μM) 0.8 μL, reverse primer (5 μM) 0.8 μL, *TransStart* FastPfu DNA Polymerase 0.4 μL, template DNA 10 ng, and ddH_2_O up to 20 μL. PCRs were performed in triplicate. The PCR product was extracted from a 2% agarose gel, purified using the AxyPrep DNA Gel Extraction Kit (Axygen Biosciences, Union City, CA, United States) according to the manufacturer’s instructions, quantified using a Quantus™ Fluorometer (Promega, Madison, WI, United States), and then sequenced on an Illumina MiSeq PE300 platform (Illumina, San Diego, CA, United States).

### Metabolite Extraction and Metabolomics Analysis

Fifty milligrams of soil sample was accurately weighed, and the metabolites were extracted using a 400 μL methanol: water (4:1, v/v) solution. The mixture was allowed to settle at –20°C and treated with a Wonbio-96c high-throughput tissue crusher (Shanghai Wanbo Biotechnology Co., Ltd., Shanghai, China) at 50 Hz for 6 min, followed by vortexing for 30 s and ultrasonication at 40 kHz for 30 min at 5°C. The samples were placed at –20°C for 30 min to precipitate proteins. After centrifugation at 13,000 *g* at 4°C for 15 min, the supernatant was transferred to sample vials for LC–MS/MS analysis (Wang and Xie, 2020).

A multivariate statistical analysis was performed using ropls (Version 1.6.2^1^) R package. Principal component analysis (PCA) using an unsupervised method was applied to obtain an overview of the metabolic data, and general clustering, trends, or outliers were visualized. All metabolite variables were scaled to unit variances prior to conducting the PCA. Partial least squares discriminate analysis (PLS-DA) was used for statistical analysis. All metabolite variables were scaled to Pareto scaling prior to conducting the PLS-DA. The model validity was evaluated from model parameters *R*^2^ and *Q*^2^, which provide information on interpretability and predictability, respectively, of the model and avoid the risk of overfitting (Wang et al., 2021). Variable importance in the projection (VIP) was calculated in the PLS-DA model, and p values were estimated with paired Student’s *t*-test on single-dimensional statistical analysis. The variables with significant differences (*p* < 0.05) and VIP values > 1 were defined as key metabolites.

### Univariate Statistical Analysis

Univariate statistical analyses were performed using SPSS 19.0 and summarized as the mean ± standard error (SE). Treatment means were compared using Tukey’s ANOVA at the 5% level of significance (*p* < 0.05).

## Results

### Content of Available Arsenic and Arsenic Fractionation in Soil

**FIGURE 1:**
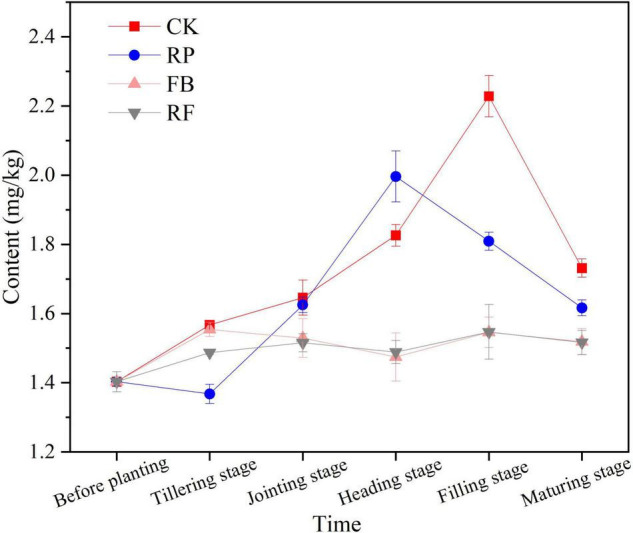
Available arsenic content in soils in different treatments at different growth stages.

Figure 1 shows the changing trend of available arsenic content in soil during the rice growth period. In the flooded paddy soil, the available As content in the soil first increased and then decreased with the growth period of the rice plants. In the CK and RP treatments without inoculation with FeOB, the content of available arsenic in soil increased greatly in the early stage of rice growth because paddy soil was flooded at that time, and the redox potential in the soil decreased, resulting in the reduction and dissolution of arsenic originally adsorbed on iron oxides and their re-release into the soil (Wu et al., 2016). The available arsenic content of soil reached the highest value, 2.228 mg/kg, at the jointing stage in CK, while in the treatments planted with rice (RP), the available As content was lower. In the FB and RF treatments inoculated with FeOB, the available arsenic content in the soil increased slightly with time, but the total content remained at the same low level as the initial value (1.487–1.554 mg/kg). The inoculation of FeOB significantly reduced the available arsenic content in paddy soil from the jointing stage to the maturity stage (*P* < 0.05). In the maturity stage, the available arsenic content of each treatment group was CK > RP > FB > RF, among which the available arsenic contents in the FB and RF treatments were 1.518 mg/kg and 1.516 mg/kg, respectively, which were 6.25–12.31% lower than the 1.731 mg/kg and 1.617 mg/kg of the CK and RP treatments, respectively.

**FIGURE 2:**
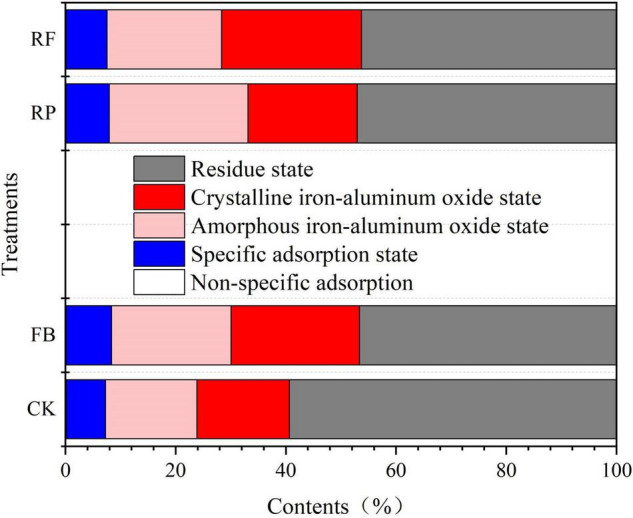
Relative distribution of different arsenic binding states of different treatment groups at the maturing stage in paddy soils.

Since the total arsenic concentration is insufficient to account for the bioavailability and toxicity of arsenic in soil (Huang et al., 2015), we used different extraction reagents to separate the arsenic components in the soil into the following binding states: (1) non-specific adsorption state, (2) specific adsorption state, (3) amorphous iron-aluminum oxide binding state, (4) crystalline iron-aluminum oxide binding state, and (5) residual state. Figure 2 shows the different arsenic fractions of soils in the rice maturation stage. In the CK, the As fraction concentration trend was residual state > amorphous iron-aluminum oxide binding state ≥ crystalline iron-aluminum oxide binding state > specific adsorption state > non-specific adsorption state. In other treatments, the As concentration in the residual state was 56.3∼82.0 mg/kg, accounting for 46∼59%; As concentration in the crystalline iron-aluminum oxide binding state was 23.2∼29.8 mg/kg, accounting for 17∼25%; As concentration in the amorphous iron-aluminum oxide binding state was 22.9∼32.7 mg/kg, accounting for 17∼25%; As concentration in the specific adsorption state was 9.55∼10.2 mg/kg, accounting for 7∼8%; and As concentration in the non-specific adsorption state was 0.15∼0.23 mg/kg, accounting for <1%. In unplanted soil, compared with the CK, FeOB inoculation (FB) increased the As fraction of the amorphous iron-aluminum oxide binding and crystalline iron-aluminum oxide binding states by 5 and 6%, respectively. However, the residual state decreased by 12% in the FB treatment compared with the CK. In rice rhizosphere soil, compared with the RP, FeOB inoculation (RF) increased the As fraction of the crystalline iron-aluminum oxide binding state by 5% but decreased the As fraction of the amorphous iron-aluminum oxide binding state by 4%.

### Arsenic Content and Speciation in Rice Plants

**FIGURE 3:**
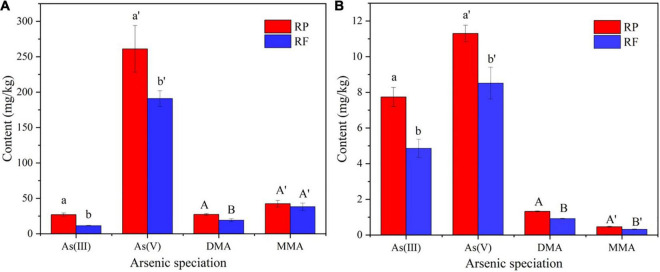
Contents of arsenic in different speciation in roots **(A)** and stems **(B)** of rice plants.

In order to explore the effect of inoculation of iron-oxidizing bacteria on the accumulation of As in rice plants, we harvested rice at mature stage and determined the arsenic speciation in the tissues. Figure 3 shows the As speciation concentrations in roots (A) and stems (B) of rice plants. Most As species in rice plants was inorganic As, while organic As accounted for lower proportions. In rice plants, the different As speciation trend was As(V) > As(III) > DMA ≥ MMA, which was in accordance with Smith (Smith et al., 2008). The contents of As(III) and As(V) in roots with FR treatment were 11.5 and 191 mg⋅kg^–1^, respectively, while those with RP treatment were 27.0 and 261 mg⋅kg^–1^, which were reduced by 57.4 and 26.8%, respectively. In the stems, with the inoculation of iron oxidizing bacteria, the contents of As(III) and As(V) decreased from 7.74 and 11.3 mg⋅kg^–1^ to 4.86 and 5.82 mg⋅kg^–1^, which were reduced by 37.2 and 48.5%, respectively. Apparently, the inoculation of iron-oxidizing bacteria significantly reduced the contents of As(III), As(V) and DMA in rice tissues.

### Microbial Community Diversity

The species diversity index is a function of species richness and evenness and is a statistic used to describe the diversity of a community. As shown in Table 2, the evenness (Shannon and Simpson) and abundance (ACE and Chao) of the microbial community decreased in the treatments with FeOB (FB and RF), indicating that the addition of FeOB caused a disturbance to the microbial community structure in the original soil, in which the niches of some kinds of microorganisms were replaced, thus reducing microbial diversity. In addition, the microbial community diversity in the rhizosphere soil under different treatments was higher than that in the unplanted rice soil because the exudates from plant roots could have provided a carbon source for soil microorganisms and could have changed the microenvironmental conditions of the rhizosphere soil. These results were consistent with the results reported by Peiffer et al. (2013), who found that the microbial community diversity in the maize rhizosphere was significantly higher than that in the surrounding soil.

**TABLE 2:** α diversity index of microbial communities in the soils.

Sample	Shannon	Simpson	Ace	Chao
RF	6.864	0.003103	5357	5334
FB	6.776	0.003557	5553	5558
RP	7.175	0.002425	7232	7248
CK	6.974	0.002699	6360	6299

**FIGURE 4:**
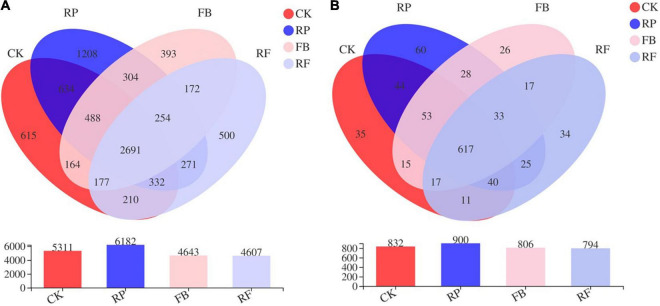
Venn diagram of the microbial community at the OUT **(A)** and genus **(B)** level.

As shown in Figure 4A, the number of microbial OTUs in the paddy soil and unplanted soil without FeOB was 5311 and 6182, respectively. The number of microbial OTUs detected in the paddy and unplanted soil inoculated with FeOB was 4643 and 4607, respectively. The number of common OTUs in the four treatment groups was 2691, accounting for 50.67, 43.53, 57.96, and 58.41% of the CK, RP, FB, and RF treatment groups, respectively. Figure 4B shows that the CK, RP, FB, and RF treatment groups contained 832, 900, 806, and 794 genera, respectively, and 617 bacterial genera were shared among all treatment groups. Compared with the treatments without inoculation of FeOB, the number of common genera in the soil with inoculation of FeOB decreased relatively, while the number of specific genera increased relatively.

**FIGURE 5:**
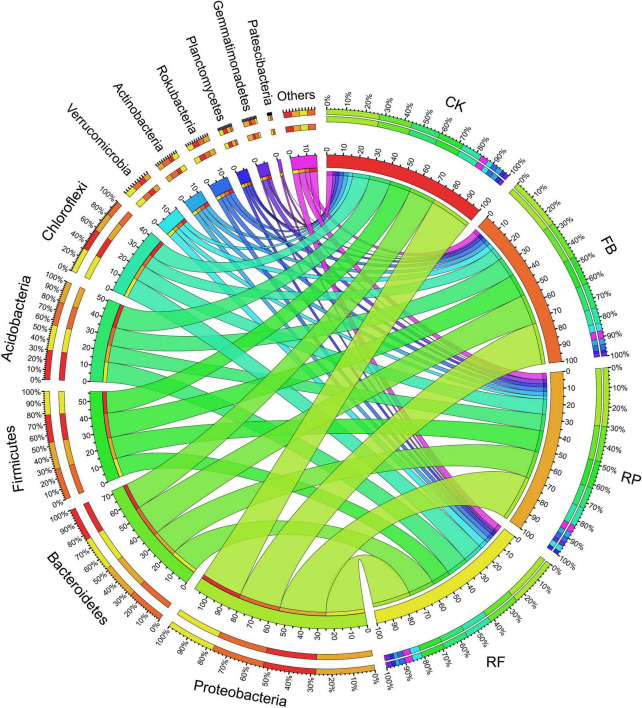
Circos diagram of microbial on phylum levels in different treatments.

**FIGURE 6:**
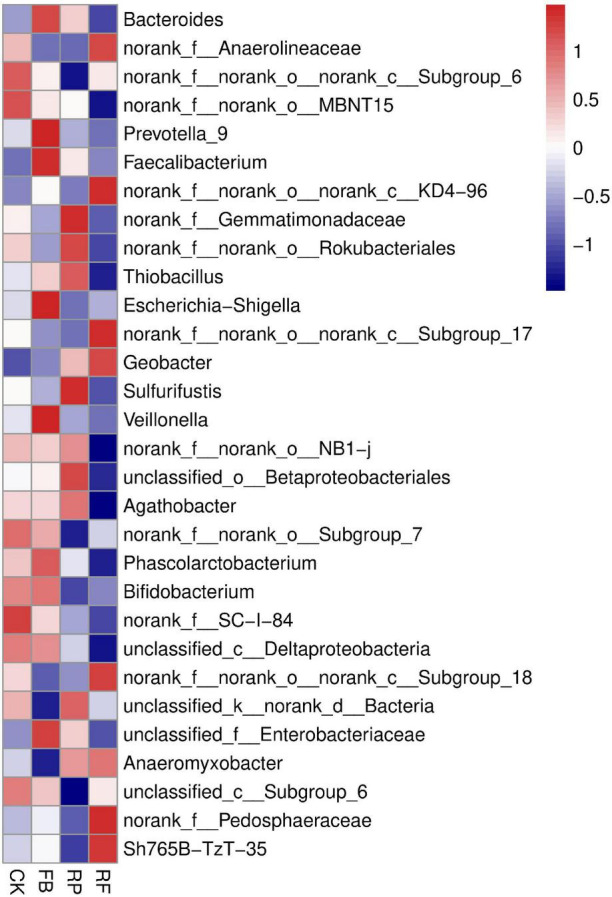
Heat map of microbial communities at the genus level.

The soil samples collected in this experiment included Proteobacteria, Bacteroidetes, Firmicutes, Acidobacteria, Chloroflexi, Verrucomicrobia, Actinobacteria, Rokubacteria, Planctomycetes, Gemmatimonadetes, and Patescibacteria, accounting for more than 95% of the total phyla (Figure 5). Among them, Proteobacteria, Bacteroidetes, Firmicutes, and Acidobacteria were the dominant phyla, and their relative abundances in the CK treatment were 27, 17, 13, and 15%, respectively. In the FB treatment, the relative abundance of Firmicutes increased by 3%, that of Bacteroides increased by 3%, and that of Acidobacteria decreased by 2%. The changing trend of microbial phyla caused by inoculation with FeOB was different in the paddy soil compared with that in the unplanted soil. In the paddy soil, the relative abundances of Proteobacteria and Firmicutes increased by 5 and 2% after inoculation with FeOB, respectively. In genus level, the relative abundance of *Bacteroides*, *Anaerolineaceae*, *Prevotella*, *Faecalibacterium*, *Gemmatimonadaceae*, *Rokubacteriales*, and *Thiobacillus* was higher (Figure 6). However, the unclassified KD4-96 (belonging to the phylum Phanerochaete), *Pedosphaeraceae*, *Geobacter* were significantly enriched after inoculation with FeOB, while the abundances of *Gemmatimonadetes* and *Rokubacteriales* etc. were lower in the treatments with FeOB inoculation.

### Soil Metabolic Profiling

**FIGURE 7:**
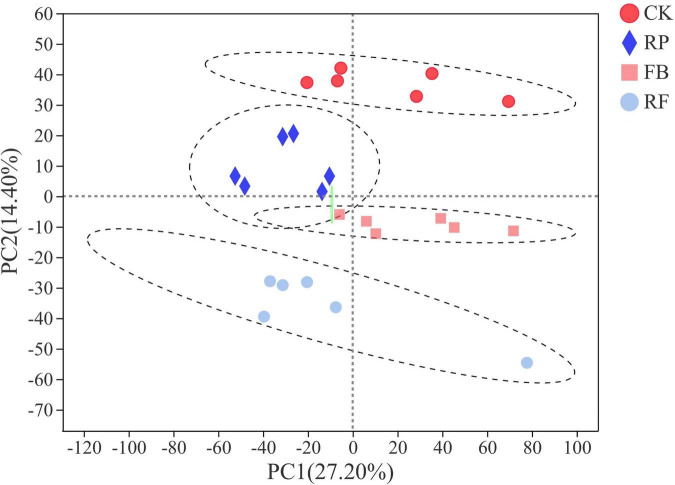
PCA scoring model of different treatments.

**FIGURE 8:**
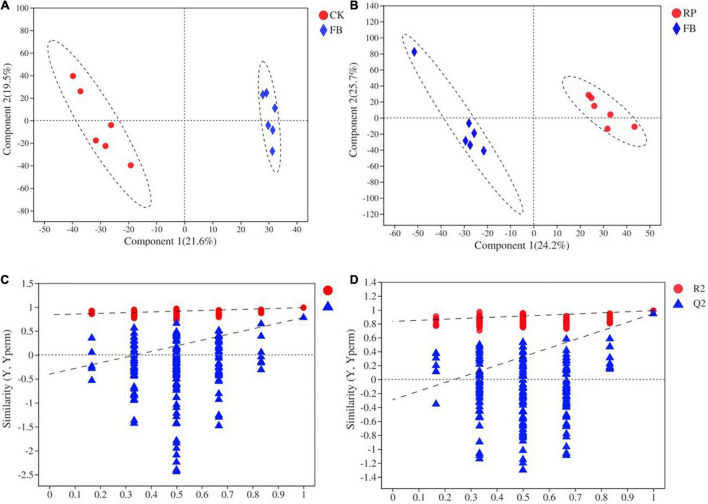
PLS-DA scoring [**(A)** CK and FB and **(B)** RP and RF] models and permutation test [**(C)** CK and FB and **(D)** RP and RF].

Soil metabolomics can detect hundreds of chemicals simultaneously and identify metabolic molecular changes that have occurred in the soil. However, current research on the effects of FeOB on soil microbial remediation generally does not include the analysis of soil metabolites. In this study, FeOB were added to the soil as *in situ* remediation agents to study the long-term effects of FeOB on soil metabolite profiling in the presence and absence of rice plants. To visualize general grouping information as a function of treatment, PCA analysis was performed (Figure 7). The results showed that except for the large deviation in the RF4 samples, the remaining samples were within the 95% confidence interval, and there was a significant difference between the two groups. However, the samples in the RP group were not completely separated due to their proximity to each other. The results showed that all samples except RF4 were within the 95% confidence interval. In Figure 7, PC1 and PC2 represented 27.20 and 14.40%, respectively, of the overall difference of the samples, and the FeOB inoculated treatments (RP and RF) and the non-inoculated treatments could be completely separated (CK and FB). In addition, whether rice was planted was considered the distinguishing condition, and the samples of the two treatments were also completely separated. And to achieve the maximum separation between soil metabolic samples from different treatment groups, partial least squares analysis (PLS-DA) with a supervised model was used to analyze the difference between two treatments. As shown in Figure 8, all 24 samples were in the confidence interval of 95%, and the group of CK, RP and FB, RF were obviously separated, which indicates that components 1 and 2 in this model can better explain the differences between groups, and the metabolite abundance among groups were significantly different. The replacement test of the model (Table 2) shows that R2X and R2Y represent the interpretation rate of the x and y matrices, and R2X(cum) and R2Y(cum) represent the cumulative interpretation rate. Q2 indicates the prediction ability of the model. The closer these three indices are to 1, the more stable and reliable the model, and Q2 > 0.5 indicates the better prediction ability of the model. The R2Y(cum) of the two PLS-DA models is close to 1, which indicates that the models agree with the real situation of samples, Q2 is greater than 0.5, and the intercept between q and the longitudinal axis is less than 0, which indicates that the models have good stability.

**FIGURE 9:**
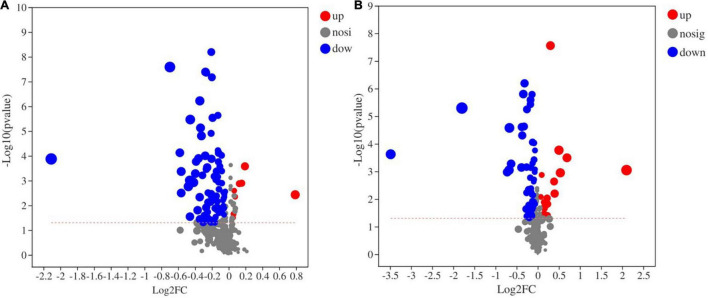
Volcano map of metabolic differences (**A:** CK vs. FB; and **B:** RP vs. RF).

**FIGURE 10:**
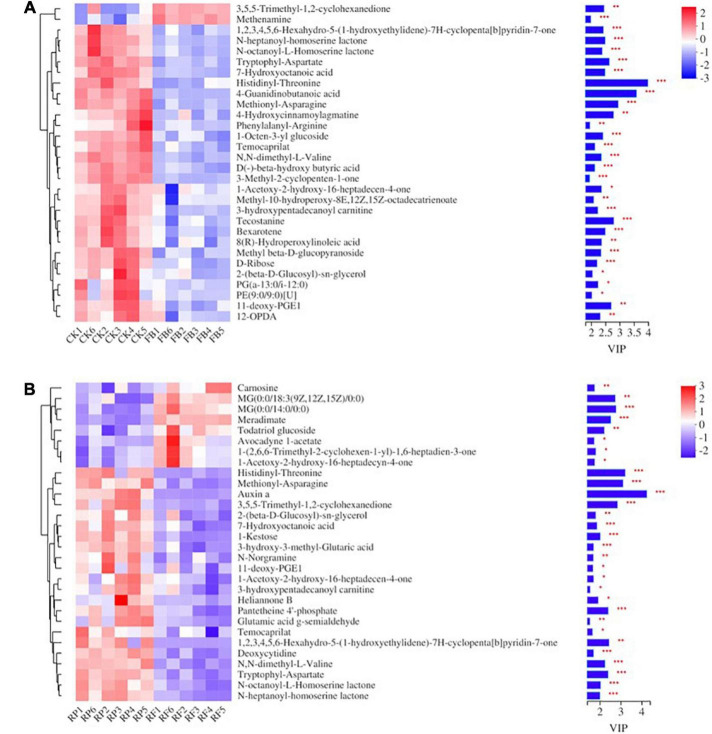
Heat map of metabolic differences of the top 30 metabolites in unplanted **(A)** and rice rhizosphere **(B)** soils.

A total of 448 metabolites were identified and semiquantified in soil samples by using non-target metabonomics based on liquid chromatography-mass spectrometry (LC–MS). A two-tailed test analysis was performed on all metabolites, and a volcano map of metabolite differences was created (Figure 9). Figure 9A shows the differential metabolites in the CK and FB, and most differential metabolites were significantly downregulated in the FB, with only a few metabolites were significantly upregulated. Notably, the inoculation of FeOB had different effects on soil metabolites in the rice rhizosphere, more metabolites were significantly upregulated in RF. According to *P* < 0.05 and VIP > 1, the differential metabolites with the top 30 VIP values are shown in Figure 10. In the treatment groups without rice planting, except for the increase in two metabolites, the abundance of most differential metabolites was downregulated with the inoculation of FeOB (Figure 10A), which indicated that FeOB might affect the metabolism of soil microorganisms, resulting in the inhibition of some metabolite synthesis pathways or the enhancement of decomposition pathways. Notably, the abundance of several lipids and lipid-like compounds was significantly downregulated with inoculation of FeOB, including fatty alcohols (1-acetoxy-2-hydroxy-16-heptadecen-4-one), fatty acid esters (3-hydroxypentadecanoyl carnitine), retinoids (bexarotene), linoleic acids and their derivatives [8(*R*)-hydroperoxylinoleic acid], fatty acyl glycoside (1-octen-3-yl glucoside) and glycerophospholipids [PG (a-13:0/i-12:0)]. In addition, the abundance of carbohydrates and their derivatives, including methyl beta-D-glucopyranoside, D-ribose, 1-octen-3-yl glucoside, sucrose and amino acid derivatives, including rhizonin A, histidinyl-threonine, tryptophyl-aspartate, temocarilat, methionyl-asparagine, aminopentanamide and others, were also downregulated with inoculation with FeOB.

In the presence of rice plants, the soil metabolite pool consists of metabolites secreted by plants and metabolites produced by microbial communities. The results showed that inoculation with FeOB also changed the distribution of soil metabolites in the rhizosphere soil. Part of the key metabolites were consistent with those in the unplanted soil (Figure 10B), including some amino acid analogs and some lipid compounds such as *N-N*-heptanoyl-homoserine lactone NE and *N*-octanoyl-L-homoserine lactone etc., which were still downregulated after inoculation with FeOB. Furthermore, some other key metabolites not found in the unplanted soil, such as pantetheine 4′-phosphate, etriol-3-glucuronide (not shown in the figure), were also downregulated. Interestingly, in contrast to the unplanted soil, more metabolite abundances were upregulated after inoculation with FeOB in the paddy-planted soil, including carnosine, MG(0:0/18:3(9Z,12Z,15Z)/0:0), MG(0:0/14:0/0:0), todatriol glucoside, avocadyyne 1-acetate, (2,6,6-trimethyl-2-cyclohexen-1-yl)-1,6-heptadien-3-one, and acetoxy-2-hydroxy-16-heptadecyn-4-one.

## Discussion

### Redistribution of Arsenic in Paddy Soil and Rice Plants by Inoculation With FeOB

The toxicity and mobility of As depend largely on its speciation, the content of total arsenic in soil is of limited significance for measuring the bioavailability of As in soil (Flynn et al., 2002). Therefore, the determination of available As content and the As fractionation in soil is of great significance to the environmental risk assessment of arsenic in soil. The available As content in this study were rank as follow: CK > RP > FB ≥ RF (Figure 1). The available As content in the treatments planted with rice (RP) was lower than CK, which was due to the oxygen secretion function of the rice root system, promoting the formation of iron plaque on the rice root surface and immobilizing the heavy metals through adsorption or coprecipitation, thus resisting the damage to the plants by the heavy metals in the soil (Wu et al., 2015). The inoculation of FeOB effectively controlled the content of available arsenic in soil and inhibit the desorption and release of adsorbed arsenic in flooded soil. It was because FeOB promoted the aggregation of Fe (II), accelerated the oxidation rate of Fe (II) and promoted the formation of iron (hydroxide) oxides under microaerobic or anaerobic conditions. It was reported that most iron (hydroxide) oxides generated from Fe(II) oxidation mediated by neutral FeOB initially exist as second-line ferrihydrite (Fe_5_HO_8_⋅4H_2_O) or amorphous iron oxyhydroxide (FeOOH) (Miot et al., 2009). These iron minerals have the characteristics of high surface activity, low crystallinity, amorphous state, and strong adsorption performance for heavy metals. However, second-line ferrihydrite was in a metastable state in the environment and was prone to conversion into mineral phases and morphological structures under the action of chemicals or microorganisms, thus further forming high-crystallinity and more stable iron minerals such as lepidocrocite, hematite, goethite and magnetite. This process was usually accompanied by the migration and release of heavy metals from the mineral surface, followed by secondary adsorption and coprecipitation.

As fractions in the soil could have been affected by the rice rhizosphere environment, including physicochemical properties (such as pH and Eh), radial oxygen loss of rice (ROL) and microbes in the rice rhizosphere. For example, Wu et al. showed that ROL could affect As transfer in soils, leading to the coprecipitation of As and Fe and increasing the As fraction of the amorphous iron-aluminum oxide binding state (Wu et al., 2015). Moreover, the activities of plant roots and microorganisms promote the oxidation of difficult-to-convert substances such as sulfide (FeAsS) and organic matter in soil (Huang et al., 2015). For example, FeOB oxidized Fe(II)to Fe(III), which accelerated the oxidation-dissolution reaction of As-containing minerals such as arsenopyrite, leading to the formation of FeAsO_4_⋅2H_2_O (Garcia-Sandchez and Alvarez-Ayuso, 2003). This process increased the As content in the iron-aluminum oxide binding state but decreased the As content in the residue state. In addition, the radial oxygen loss of rice might stimulate microbial activities in rhizosphere soil and further affect the behavior of As.

Previous studies (Wenzel et al., 2001; Smith et al., 2008) have shown that As in soil is closely related to amorphous or crystalline iron-aluminum oxides. The As component in the non-specific adsorption state, specific adsorption state, and amorphous iron-aluminum oxide binding state was considered to constitute the main part of bioavailable As (Tang et al., 2007), whereas the crystalline iron-aluminum oxide binding state and residual state were relatively stable. Therefore, the As component associated with the amorphous iron-aluminum oxide binding state was a key part in controlling bioavailability. In these results, the inoculation of FeOB increased the amorphous iron-aluminum oxide binding state and crystalline iron-aluminum oxide binding state of As in the soil without rice planting. In the rhizosphere soil, the inoculation of FeOB promoted the transformation of the amorphous iron-aluminum oxide binding state of As to the crystalline iron-aluminum oxide binding state, which indicated that the application of FeOB in soil resulted in the redistribution of arsenic speciation in soil and effectively reduced the bioavailability of As.

With the inoculation of FeOB, different As speciation, especially As(V), As(III) and DMA, were significantly decreased in both stems and roots. FeOB could have promoted the formation of rhizosphere iron (hydrogen) oxide and the formation of iron plaque, which could have sequestered As on rice roots. This has potential for As remediation in soils and decreasing As accumulation in rice plants. Moreover, other studies also showed that with the addition of FeOB to paddy fields, the As and Cd concentrations in different parts of rice significantly decreased (Dong et al., 2016; Xiao et al., 2020).

### Changes of Soil Microbial Community Structure After Inoculation With FeOB

These results of microbial community diversity indicated that inoculation with FeOB changed the microbial composition in the microdomain of paddy soil and could significantly affect the growth of some microorganisms, resulting in significant differences in microbial communities (Figure 4). At the phylum level, with the inoculation of iron-oxidizing bacteria, Proteobacteria, Bacteroidetes, and Firmicutes were increased in the unplanted and rice rhizosphere soils (Figure 5). The enrichment of Proteobacteria, Bacteroidetes, and Firmicutes was consistent with the previously reported microaerophilic FeOB studies. In the study of enrichment and subculture of micro-aerobic iron-oxidizing bacteria in paddy soil by Chen et al. (2016) and Li et al. (2019), enrichment of some bacteria with iron oxidation function was found, and most of them belong to Proteobacteria, Bacteroides, and Firmicutes, accounting for more than 90% of microbial community, which indicates that the inoculation of *Ochrobactrum* strain promotes the enrichment of iron oxidation-related microorganisms in soil in this study. Moreover, the *Ochrobactrum* strain in our study, belongs to Proteobacteria. Although the abundance increase of Proteobacteria was detected in the results of microbial communities, *Ochrobactrum* sp. was not detected as dominant genus at the genus level, which might be since after a long period of culture, *Ochrobactrum* sp. competed with indigenous microorganisms, and have not developed into the dominant genus or the universal primers used for PCR amplification in this study had no specificity for 16S rRNA genes of Ochrobactrum (Wang et al., 2009).

In the results of the genus level (Figure 6), Girardot et al. (2020) reported that KD4-96 was a heavy metal-resistant bacterium, and it may play an important role in the biotransformation of heavy metals. Cao et al. (2020) found that *Pedosphaeraceae* had a significant positive correlation with the accumulation of Pb and Cd in hyperenriched plants, but the behavioral mechanism was not elucidated. The enrichment of *Pedosphaeraceae* might indicate that inoculation with FeOB reduced the toxicity of heavy metals in soil (Cao et al., 2020). The abundances of *Gemmatimonadetes* and *Rokubacteriales* were lower in the FeOB inoculation treatments. *Gemmatimonadetes* is a beneficial bacterium essential for phosphorus dissolution, microbial nitrogen metabolism and soil respiration (Chen et al., 2020) and widely exists in farmland soil. Similarly, studies have shown that Rokubacteriales is a type of bacteria with nitrogen respiratory potential (Becraft et al., 2017). These results indicated that the relevant carbon and nitrogen metabolic pathways were relatively weakened in the treatment with FeOB after 105 days of inoculation. It was speculated that the inoculation of nitrate dependent, FeOB at an early stage stimulated the process of iron oxidation coupled with nitrogen reduction and accelerated the consumption of nitrogen sources in the soil, thus weakening the nitrogen metabolic pathway at the mature stage. Furthermore, the abundance of *Geobacter*, which is related to iron-reducing bacteria, was higher in the rhizosphere soil than in the unplanted soil, and the abundance was highest in the RF, suggesting that the action of oxygen secretion from the rice rhizosphere and FeOB jointly promoted the iron redox cycle in the soil.

### Regulatory Mechanism of FeOB in Soil From the Perspective of Metabonomics

The structure and function of soil ecosystems are very complex, and their key biogeochemical cycles are driven by a variety of factors, including soil animals, microorganisms, extracellular enzymes, and plants. Soil metabolites can be considered phenotypic to changes in soil microbial community activity, as changes at the biological and enzyme levels will ultimately be manifested as changes in the metabolite profiles. In addition, plants can also actively regulate the distribution of rhizosphere secretions. Swenson et al. (2015) first proposed the “soil metabolomics” method to analyze soil organic matter reservoirs, and the changes experienced by organisms and enzyme levels are manifested as changes in metabolic profiling to propose reasonable assumptions for changes in soil biochemistry. In this study, FeOB may directly or indirectly interact with soil organic matter and microorganisms and thus affect biochemical processes and metabolic pathways in soil. The secreted intracellular and extracellular metabolites of soil microorganisms constitute the soil metabolite pool, and monitoring soil metabolites could indirectly reflect the changes in microbial metabolism.

In unplanted soil (Figure 10A), (R)-Hydroperoxylinoleic acid is a linoleic acid derivative that can be produced by some fungi through linoleic acid metabolism (Brodowsky et al., 1992; Brodowsky and Oliw, 1993), while PG(a-13:0/i-12:0) is a glycerophospholipid that is not only an important component of biofilms but also involved in protein recognition and signal transduction by the cell membrane. Oxylipins are a type of lipid oxide signal that regulates many physiological processes, such as growth and development, defense responses to pathogens and herbivores, and abiotic stresses (Wang et al., 2020). 12-OPDA, a metabolite of the allene oxide synthase (AOS) pathway, is produced by the oxidation of α-linolenic acid by 13-lipoxygenase, followed by the formation of unstable alkylene oxide through AOS action and subsequent cyclization by alkylene oxide cyclase (Stenzel et al., 2012), which has been reported to arise under stress in response to a variety of environmental changes. Moreover, fatty acids and their derivatives, such as stearidonic acid, 9(S)-HODE, 9-OxoODE, and prostaglandin J2, also showed significant differences between the FB and CK. According to the functional pathway of the KEGG pathway, these unsaturated fatty acid cascades could produce a variety of endogenous signaling molecules to regulate a variety of biological processes (Milne et al., 2005). D-ribose has been reported as the structural skeleton of purine and pyrimidine in genetic material and pentose precursor of some amino acids and is a component of many cofactors (especially adenosine triphosphate), playing a key role in energy metabolism (Croci et al., 2011; Maifiah et al., 2017). The reduction of these carbohydrates and amino acids might affect the normal microbial activities in the soil. The downregulation of carbohydrate abundance in soil might be due to the increase in carbohydrate molecular consumption caused by the addition of FeOB or due to the changes in the composition of microbial communities, which affect the carbon cycle in soil. A series of studies showed that exopolysaccharides and proteins secreted by bacteria are likely to provide nucleation sites for mineralization in the process of metal ion precipitation (Chan et al., 2004, 2009; Miot et al., 2009), inducing the accumulation of metal ions and the formation of different mineral phases. Huang et al. (2015) demonstrated that polysaccharides, such as dextran, chitosan, gelatine, and protein, could serve as nucleation sites and regulate the biomineralization process (Sun and Huang, 2006; Huang and Sun, 2007). The results of this study showed that the abundance of carbohydrates and amino acid analogs was downregulated to a certain extent after inoculation with FeOB. Combined with the results of the increase in the amorphous iron-aluminum oxide binding state and crystalline iron-aluminum oxide binding state of As in paddy soil, it could be speculated that the decrease in the abundance of polysaccharides and protein was due to the sampling time at the maturity stage of rice. A period after inoculation with FeOB promoted the utilization of polysaccharides, amino acids, and other substances in the soil during the growth stage of rice, as well as the occurrence of biomineralization, which in turn led to the downregulation of protein polysaccharide abundance at the maturity stage.

In rice rhizosphere soil (Figure 10B), Carnosine was reported as an endogenous dipeptide present in different tissues that inhibits lipid peroxidation and protein carbonylation (Kang et al., 2002; Aldini et al., 2005; Aydin et al., 2010; Kalaz et al., 2014). Carnosine functions as a free radical scavenger, a membrane protector, and a transition metal-chelating agent in organisms and has superoxide dismutase-like activity (Boldyrev et al., 2010) and is capable of neutralizing lipid peroxidation. The increase in carnosine abundance was conducive to resisting the destructive effects of oxidant pollutants. In addition, the abundance of most lipids and their derivatives in soil was upregulated by RP treatment, such as MG(0:0/18:3(9Z,12Z,15Z)/0:0), MG(0:0/14:0/0:0), todatriol glucoside, avocadyyne 1-acetate, (2,6,6-trimethyl-2-cyclohexen-1-yl)-1,6-heptadien-3-one, and acetoxy-2-hydroxy-16-heptadecyn-4-one. The upregulation of lipid compounds indicated that the inoculation of FeOB affected the lipid metabolic pathway in the soil planted with rice, but the abundance of lipid compounds showed the opposite change trend to that in the unplanted soil, which also indicated that the occurrence of lipid peroxidation in the soil was inhibited to a certain extent. Moreover, pantetheine 4’-phosphate was reported to be capable of synthesizing CoA, which is involved in many metabolic pathways, including the citric acid cycle, fatty acid biosynthesis as the coenzyme of acyltransferase (Rath et al., 2018). Some studies have shown that pantothenic acid and its derivatives have a protective effect on cell and tissue damage and are related to resistance to peroxidation damage (Slyshenkov et al., 1995). Besides, the abundances of *N-N*-heptanoyl-homoserine lactone NE and *N*-octanoyl-L-homoserine lactone were downregulated. Other studies reported that homoserine internal lipids also affected plant cells. If homoserine lactone exists around plants, plants might change gene expression, protein levels, and growth status and enhance their defense response (Mathesius et al., 2003; Miao et al., 2012; Zhao et al., 2016).

In summary, FeOB still downregulated the abundance of some metabolites in the rice rhizosphere soil, indicating that inoculation of FeOB caused a disturbance to the microbial community structure in the soil. However, there were a few upregulated metabolites, especially some compounds related to lipid metabolism. It was speculated that the coregulation of plant root exudates and soil microorganisms might enhance the defense ability of the soil-microorganism-plant system against peroxidation damage. However, the potential mechanism of these changes, including the role of plants, and whether such regulation is active or passive are still unclear.

## Conclusion

The iron-oxidizing strain (*Ochrobactrum* sp. EEELCW01) was inoculated into As polluted paddy soil as a microbial agent, which could effectively reduce As bioavailability. The content of soil available As in the treatments (FB, RF) inoculated with iron oxidizing bacteria was decreased by 6.25–12.31% compared with that in the treatments (CK, RP) not inoculated. The inoculation of iron oxidizing bacteria also increased the proportion of As in the iron-aluminum oxide binding fraction, and significantly reduced As accumulation in rice tissues. The abundances of KD4-96, Pedosphaeraceae and other bacteria in soils increased with the inoculation of iron oxidizing bacteria. These bacteria had heavy metal resistance and could reduce the toxicity of heavy metals in soil through the biotransformation. However, the abundance of Gemmatimonadetes and Rokubacteriales decreased relatively, which was related to the consumption of a large amount of N-derived substances in the early stage. Soil metabolites could reflect the changes in microbial metabolic process. In the unplanted soil, the reduction of most polysaccharides and protein substances might be due to the promotion of iron oxide bacteria to metabolize and decompose polysaccharides, amino acids and other substances in the soils. In the rhizosphere soils, the abundance increases of Carnosine and some lipid substances including MG (0:0/14:0/0:0) and Pantetheine 4′-phosphate indicated that the defense ability of soil-microorganism-plant system against peroxidation damage was improved.

## Data Availability Statement

The original contributions presented in the study are included in the article/supplementary material, further inquiries can be directed to the corresponding author/s.

## Author Contributions

ZQ: experiment, data curation, formal analysis, software, visualization, writing – review and editing. CW: methodology, resources, writing – review and editing. WP: funding, conceptualization, and resources. XX and LX: experiment and data curation. WL: review and editing. All authors contributed to the article and approved the submitted version.

## Conflict of Interest

The authors declare that the research was conducted in the absence of any commercial or financial relationships that could be construed as a potential conflict of interest.

## Publisher’s Note

All claims expressed in this article are solely those of the authors and do not necessarily represent those of their affiliated organizations, or those of the publisher, the editors and the reviewers. Any product that may be evaluated in this article, or claim that may be made by its manufacturer, is not guaranteed or endorsed by the publisher.

## Footnotes

1
http://bioconductor.org/packages/release/bioc/html/ropls.html
